# Minimally Invasive Surgery for Sternoclavicular Joint Infection with Osteomyelitis, Large Abscesses, and Mediastinitis

**DOI:** 10.1155/2022/9461619

**Published:** 2022-10-22

**Authors:** Hideki Ota, Hirotaka Ishida, Hidekazu Matsumoto, Tomoharu Ishiyama

**Affiliations:** ^1^Department of Surgery, Yamagata Prefecture Shinjo Hospital, Japan; ^2^Department of Surgery, Tohoku University Graduate School of Medicine, Japan

## Abstract

**Background:**

Sternoclavicular joint infections require en bloc resection for radical cure; however, this aggressive procedure may result in multiple adverse events. Therefore, performing minimally invasive surgery is desirable. In this report, we describe a case of sternoclavicular joint infection complicated by osteomyelitis, large abscesses, and mediastinitis that was successfully treated with incision and drainage. *Case Presentation*. A 42-year-old man with no medical history presented to our hospital with complaints of painful swelling in the left chest wall and acute dyspnea. Computed tomography revealed arthritis of the left sternoclavicular joint, osteomyelitis of the clavicle and sternum, anterior mediastinitis, and abscesses in the neck, chest wall, and retrosternal and extrapleural spaces. Gram staining of the aspirated pus revealed clusters of gram-positive cocci. A diagnosis of *Staphylococcus aureus* sternoclavicular joint infection with locoregional spread was made. Emergency surgery was performed following adequate resuscitation. A skin incision was made in the second intercostal space. The joint capsule was widely opened, necrotic tissue was curetted, and closed suction drains were placed in the abscess cavities and connected to a negative pressure system. The wound was then closed using primary sutures. The postoperative course was uneventful. Methicillin-sensitive *Staphylococcus aureus* was cultured from the pus. The patient was discharged on postoperative day 14. Osteomyelitis worsened within a few weeks after surgery but recovered with wound management and six weeks of antibiotic therapy. The patient has had no recurrence of infection for two years.

**Conclusions:**

Incision and drainage proved to be an effective minimally invasive surgical treatment for sternoclavicular joint infection with osteomyelitis, large abscesses, and mediastinitis caused by methicillin-sensitive *Staphylococcus aureus*.

## 1. Introduction

Sternoclavicular joint infection is a rare form of septic arthritis caused by infectious seeding via the hematogenous route or by direct contiguous spread [1–3]. The infection can spread to surrounding structures via the lymphatic vessels or by direct extension [4, 5], causing serious complications, such as osteomyelitis (56%), abscess formation (25%), and mediastinitis (13%) [1]. Treatment of sternoclavicular joint infections with serious complications usually requires en bloc resection of the affected joint and surrounding structures [1–14]. However, this aggressive procedure carries the risk of adverse wound events, large chest wall defects, and prolonged hospital stays [1–3, 11]. Therefore, performing minimally invasive surgery is desirable [9, 10]. Herein, we report a case of sternoclavicular joint infection with serious complications that was successfully treated with incision and drainage.

## 2. Case Presentation

**Figure 1:**
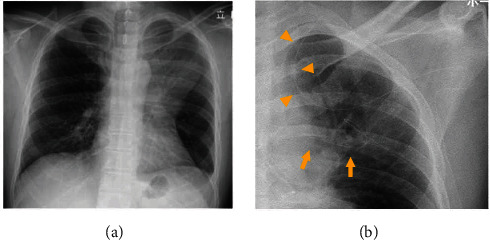
Chest radiography: (a) preoperative chest radiograph demonstrated a mass shadow in the left upper and middle lung fields; (b) postoperative chest radiography on the day of surgery showed drains in the neck and chest wall abscesses (arrowheads) and the retrosternal and extrapleural abscesses (arrows).

**Figure 2:**
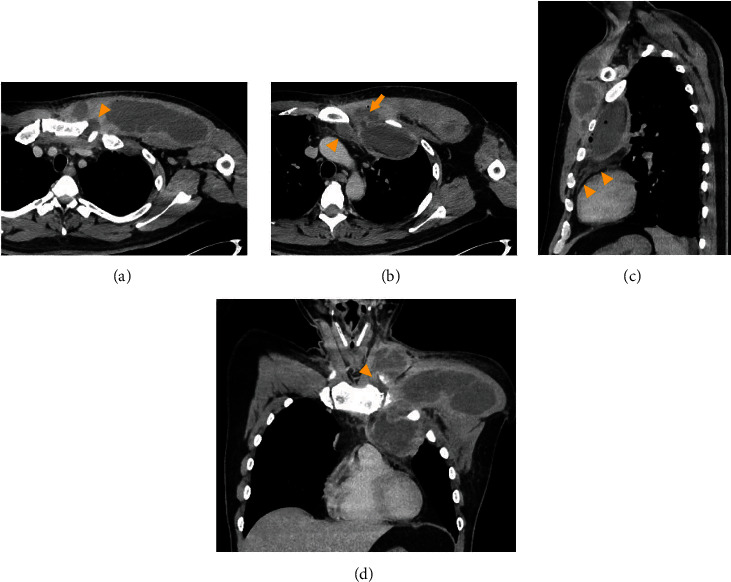
Contrast-enhanced chest computed tomography images in axial (a, b), sagittal (c), and coronal planes (d) on admission: (a) the infected joint enlarged the joint space (arrowhead); (b) the chest wall abscess was connected to the retrosternal abscess (arrowhead) and the extrapleural abscess via the first intercostal space (arrow); (c) the retrosternal abscess was complicated by anterior mediastinitis (arrowheads); (d) pyogenic arthritis of the sternoclavicular joint (arrowhead) involved the neck, chest wall, and extrapleural space.

**Figure 3:**
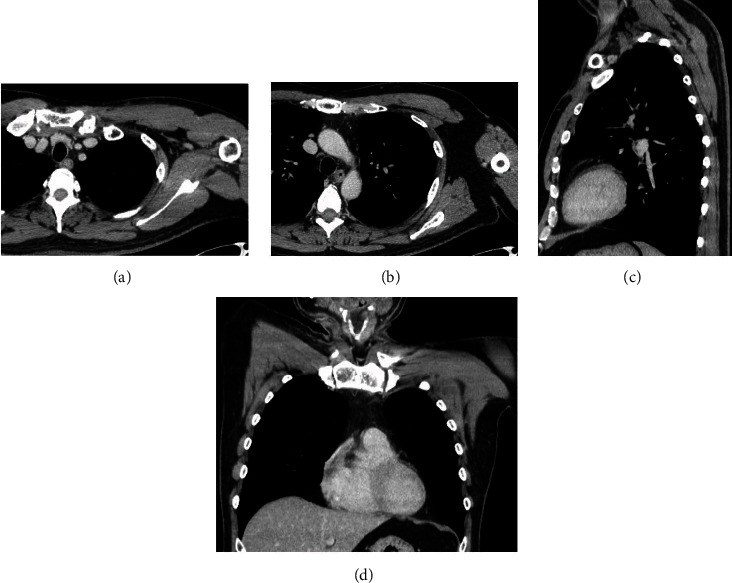
Contrast-enhanced chest computed tomography images in axial (a, b), sagittal (c), and coronal planes (d) four months after surgery.

A 42-year-old man presented to the hospital with a chief complaint of pain, swelling, and redness of the left chest wall, as well as acute dyspnea. The patient's medical history was unremarkable. He suffered blunt trauma to the left chest wall two weeks prior to presentation. Physical examination revealed a blood pressure of 98/60 mmHg, heart rate of 110 beats/min, respiratory rate of 42/min, oxygen saturation of 93% on room air, and body temperature of 38.3°C. He was drowsy with a Glasgow Coma Scale score of 13. The movement of the left shoulder was restricted. The blood test revealed a white blood cell count of 16600/*μ*L (80% neutrophils) and a C-reactive protein level of 14.34 mg/dL. Chest radiography showed a mass shadow in the left upper and middle lung fields (Figure 1(a)). Chest computed tomography (CT) revealed an enlarged left sternoclavicular joint; abscesses in the neck, chest wall, and retrosternal and extrapleural spaces (Figure 2); anterior mediastinitis (Figure 2); and osteomyelitis of the clavicle and sternum (Figure 3). Gram staining of the aspirated pus revealed gram-positive cocci in clusters and polymorphonuclear leukocytes. The sequential organ failure assessment score increased by five points from the preinfection baseline.

A diagnosis of *Staphylococcus aureus* sternoclavicular joint infection with osteomyelitis and multiple large abscesses was made. The patient was resuscitated for imminent septic shock. Intravenous antibiotic therapy with imipenem/cilastatin (2 g/day) and clindamycin (1200 mg/day) was commenced. Blood cultures were obtained. Transthoracic echocardiography revealed no significant findings of infective endocarditis due to *Staphylococcus aureus* bacteremia. Following adequate resuscitation, emergency surgery was performed under general anesthesia. A 10 cm skin incision was made in the second intercostal space. The joint capsule was widely opened, and necrotic tissue was curetted using a spoon-shaped instrument. The retrosternal and extrapleural abscesses were aspirated through the second intercostal space incision. After irrigation with saline solution, closed suction drains were placed in the abscess cavities and connected to a negative pressure system (SB VAC Super-Smooth, SB-Kawasumi Laboratories, Inc., Japan) (Figure 1(b)). The wound was closed using 3-0 nylon sutures.

**Figure 4:**
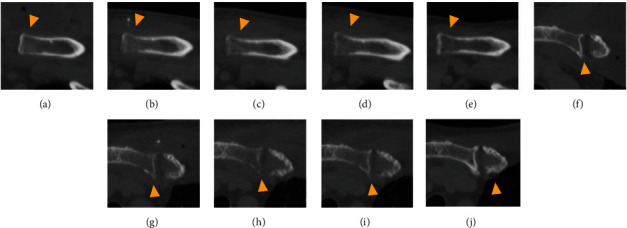
Chest computed tomography images with bone window: (a, f) on admission; (b, g) one week postoperatively; (c, h) two weeks postoperatively; (d, i) three weeks postoperatively; (e, j) four months postoperatively. Osteomyelitis (arrowhead) of the clavicle (a–e) and sternum (f–j) worsened within a few weeks after surgery but recovered within four months after surgery.

The postoperative course was uneventful. Methicillin-sensitive *Staphylococcus aureus* was cultured from the aspirated pus. Based on the culture susceptibility results, the antibiotic therapy was changed to cefazolin (4 g/day), which was administered intravenously until discharge on postoperative day 14. Thereafter, ampicillin (750 mg/day) and ampicillin/sulbactam (1,125 mg/day) were administered orally for six weeks. Cultures of the drain fluid were negative, and the drains were subsequently removed on postoperative day 12. A blood test on postoperative day 14 showed that the white blood cell count and CRP levels were within normal limits. The patient had no restrictions on shoulder movement at discharge. The sternoclavicular joint infection was cured four months after surgery (Figure 3). Osteomyelitis on CT images worsened a few weeks after surgery but recovered within four months postoperatively (Figure 4). The patient has had no recurrence of infection for two years.

## 3. Discussion

In the present case, incision and drainage was effective for the treatment of sternoclavicular joint infection with serious complications. Deep abscesses are usually treated initially with incision and drainage [9, 10]. If incision and drainage is ineffective, debridement or resection is added as curative treatment [9, 10]. However, this treatment strategy is rarely employed in sternoclavicular joint infections [1–14]. Surgical treatment for sternoclavicular joint infections is based on expert opinion and small case series owing to the limited number of cases published in the literature [3, 11, 15]. En bloc resection is recommended as the first-line treatment for sternoclavicular joint infections [1–14]. Therefore, en bloc resection is performed in 85% of sternoclavicular joint infections, debridement in 10%, and incision and drainage in 5% [14].

Incision and drainage is less invasive than debridement and en bloc resection [3, 9, 10]. The procedure varies in the literature but typically involves incision of the affected joint, opening of the joint capsule, curettage of the necrotic tissue, and drainage of the pus from the cavity [9, 10]. Incision and drainage may be sufficient for joints with limited infection but rarely resolves infections that have spread to surrounding structures [2, 3, 6–11, 16]. Therefore, incision and drainage is currently recommended as initial treatment for patients with poor general conditions [9, 11]. In this case, however, we performed incision and drainage as a curative treatment for a healthy patient with no past medical history by appropriately addressing the following three points.

First, sternoclavicular joint infections caused by *Staphylococcus aureus* can be treated with incision and drainage [9, 15]. Pyogenic arthritis of the sternoclavicular joint is mostly caused by bacterial infection [1–3]. Culture specimens are obtained from infected joints by needle aspiration or surgery, with culture-positivity rates of 77% and 36%, respectively [1]. The predominant pathogen in the general population is *Staphylococcus aureus*, which accounts for 49% of culture-positive infections [1–3, 14], as was the case in our patient. Other pathogens showing gram-positive cocci are *Streptococcus species*, accounting for 12% of cases [1]. *Staphylococcus aureus* arthritis often causes periarticular abscesses that respond well to incision and drainage [9, 15]. However, when the abscess extends to the neck or chest wall, the efficacy of incision and drainage is controversial [14, 15]. The large abscesses in this case had a clear septal formation with the surrounding structures, and pus could easily be drained through the incision.

Second, patients with retrosternal abscesses should be treated with careful attention to sepsis [17, 18]. Bacteremia occurs in 62% of patients with sternoclavicular joint infection [1]; however, sepsis is relatively rare, with a mortality rate of 8-15% [3]. On the other hand, retrosternal abscesses are more likely to cause sepsis, with a high mortality rate of 60%, reported by a study in which three of the five patients who underwent surgical drainage for retrosternal abscesses died of septic shock [17]. Resuscitation for sepsis was performed adequately before surgical drainage in our patient's case. The intercostal approach allowed for adequate surgical drainage of the retrosternal abscess without causing septic shock, empyema, or mediastinitis. Reports of surgical drainage in previous studies included en bloc or partial resection of the joint [18].

Third, wound management can prevent postoperative progression of osteomyelitis [3, 9, 18]. Treatment of osteomyelitis generally requires joint resection or bone debridement because of the poor ability of bone to eliminate infection [9, 13, 18]. Incision and drainage may be an option for minimal osteomyelitis [9], but it should be noted that osteomyelitis remains the major cause of the recurrence of infection after incision and drainage [13]. Osteomyelitis is exacerbated postoperatively by open wounds and fluid retention [11, 18]. Therefore, in this case, the incision was made away from the joint, the effusion was removed by closed suction drainage, and the wound was closed with primary sutures. To our knowledge, this is the first report demonstrating the efficacy of closed suction drainage for deep abscesses caused by sternoclavicular joint infection. CT scans were helpful in assessing postoperative osteomyelitis changes and showed improvement after two postoperative weeks. Antibiotic therapy should be continued for six weeks in cases of osteomyelitis [1].

In the present case, the patient was scheduled for reoperation if the cultures confirmed methicillin-resistant Staphylococcus aureus (MRSA). In sternoclavicular joint infections, approximately 15% of *Staphylococcus aureus* are methicillin-resistant [14]. Of the 23 patients with MRSA sternoclavicular joint infections in the literature, only one patient was effectively treated by incision and drainage [10, 14]. Therefore, en bloc resection or debridement may be a better treatment option for patients with MRSA infection [12]. Patients with risk factors for MRSA infection should receive vancomycin or daptomycin as the initial antibiotic therapy [1, 12].

## 4. Conclusion

Incision and drainage proved to be an effective minimally invasive surgical technique for the treatment of sternoclavicular joint infection with osteomyelitis, large abscesses, and mediastinitis caused by methicillin-sensitive *Staphylococcus aureus*.

## Data Availability

The dataset supporting the conclusions of this article is included within the article.

## Abbreviations

CT: Computed tomography
MRSA: Methicillin-resistant Staphylococcus aureus.
